# Schulgesundheitsfachkräfte in Deutschland – Modellprojekte und Verstetigungen

**DOI:** 10.1007/s00103-025-04104-7

**Published:** 2025-08-19

**Authors:** Franka Metzner-Guczka, Susanne Heumann-Schoop, Daniel Mays, Silke Pawils

**Affiliations:** 1 Institut und Poliklinik für Medizinische Psychologie, Universitätsklinikum Hamburg-Eppendorf, Martinistr. 52, 20246 Hamburg, Deutschland; 2 Fachbereich Erziehungswissenschaft, Universität Bielefeld, Bielefeld, Deutschland; 3 Professur für Pädagogik im Förderschwerpunkt Emotionale und soziale Entwicklung, Institut für Sonderpädagogik, Fakultät I, Pädagogische Hochschule Freiburg, Freiburg im Breisgau, Deutschland

**Keywords:** Schulgesundheitsfachkraft, Gesunde Schule, Gesundheitliche Versorgung, Bildungsgerechtigkeit, School nurse, Healthy school, Healthcare, Educational equity

## Abstract

Schulgesundheitsfachkräfte (SGFK, engl. School Nurses) leisten einen wichtigen Beitrag zur Förderung und zum Schutz der Gesundheit von Kindern und Jugendlichen im schulischen Umfeld. Sie sind spezialisierte Pflegefachkräfte mit Zusatzqualifikation, die eine verbindende Rolle zwischen Schul- und Gesundheitssystem übernehmen. Zu ihren Aufgaben zählen die gesundheitliche Betreuung chronisch kranker Kinder und Jugendlicher, die Früherkennung psychischer Auffälligkeiten, Gespräche mit Eltern, gesundheitsbezogene Aufklärung, die Unterstützung beim Zugang zu Gesundheits- und Hilfesystemen sowie die Übernahme einer Vertrauensfunktion.

In Deutschland ist die gesundheitliche Versorgung von Kindern und Jugendlichen an Schulen bislang unzureichend. Inwieweit SGFK diese Versorgungslücke schließen können, wurde im Rahmen einzelner Modellprojekte geprüft. Dabei kamen SGFK an Regelschulen zum Einsatz und ihre Wirkung auf gesundheitliche Outcomes von Schülerinnen und Schülern wurde evaluiert. Obwohl die Ergebnisse dieser Projekte vielversprechend sind, fehlt es bislang an einer flächendeckenden Implementierung von SGFK im deutschen Schulsystem.

Der vorliegende Artikel gibt einen Überblick über den aktuellen Stand des Einsatzes von SGFK an deutschen Schulen. Die Ergebnisse einer Recherche werden vorgestellt, der zufolge Modellprojekte bislang in 8 Bundesländern durchgeführt und teilweise verstetigt wurden. Der Artikel beleuchtet die zentrale Rolle von SGFK bei der Erfüllung vielfältiger gesundheitsbezogener Bedarfe von Kindern und Jugendlichen und stellt internationale Evidenz zur Wirksamkeit ihres Einsatzes dar. Abschließend wird diskutiert, weshalb SGFK trotz ihres Potenzials bislang nicht flächendeckend etabliert sind und wie ihre Einbindung ins deutsche Schulsystem gelingen kann.

## Abstract (en)

School nurses have an important contribution to make in promoting and protecting the health of children and adolescents in schools. They are specialized healthcare professionals with additional qualifications who act as a link between the school and the healthcare system. Their responsibilities include providing medical care for chronically ill children and adolescents, early detection of mental health problems, talking with parents, providing health-related information and support in accessing health and welfare services, and filling the role of a trusted confidant.

Healthcare in German schools is still inadequate. The extent to which school nurses can close this supply gap was examined in selected pilot projects. For this purpose, school nurses were deployed in mainstream schools and their impact on students’ health outcomes was evaluated. Although results from these projects are promising, there is still no nationwide implementation of school nurses in the German school system.

This article provides an overview of the current status of school nurses deployed in German schools. The presented study results show that model projects have been carried out in eight federal states to date, some of which will be continued. The article highlights the central role of school nurses in meeting the diverse health-related needs of children and adolescents and presents international evidence demonstrating the effectiveness of their use. Finally, it discusses why school nurses have not yet been established across the board despite their potential and how they can be successfully integrated into the German school system.

## Einleitung

Schulgesundheitsfachkräfte (SGFK, engl. School Nurses) engagieren sich weltweit für die Förderung und den Schutz der Gesundheit von Schülerinnen und Schülern. Sie sind zusätzlich qualifizierte Pflegefachkräfte, die sowohl im Schul- als auch im Gesundheitssystem tätig sind. Sie verfolgen das Ziel, Schulen für Schülerinnen und Schüler sowie für Lehrkräfte zu einem gesundheitsfördernden Umfeld zu machen [1]. Die Tätigkeiten der SGFK sind vielfältig: Sie umfassen die gesundheitliche Betreuung von chronisch kranken Kindern und Jugendlichen, die Früherkennung von psychischen Auffälligkeiten, Elterngespräche und Gesundheitsaufklärung, aber auch die Unterstützung bei der Anbindung ins Gesundheits- bzw. Hilfesystem (Case-Management) und nicht zuletzt die Rolle als Vertrauensperson [1]. Die Qualifikation von SGFK umfasst eine pflegerische Ausbildung, die durch eine Zusatzqualifikation im Bereich schulischer Gesundheitsförderung und -versorgung erweitert wird [2].

Das breite Spektrum an Aufgaben und Zuständigkeiten von SGFK dient dem übergeordneten Ziel, die Schule zu einem gesundheitsfördernden Lernort zu machen. Dieses Ziel wird durch 3 zentrale Teilziele konkretisiert [3]:

1. Gesundheitskompetenz erhöhen,
2. Gesundheitsversorgung anbieten,
3. Gesundheitsförderung umsetzen.

**Abb. 1:**
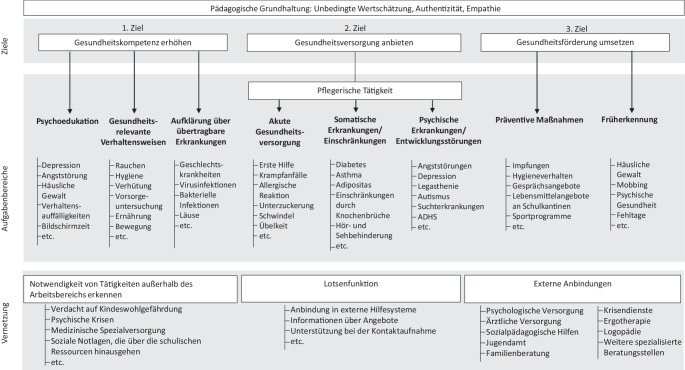
Ziele, Interventionsbereiche und Vernetzungstätigkeiten von Schulgesundheitsfachkräften (SGFK; *Asterisk* pflegerische Tätigkeiten). *ADHS* Aufmerksamkeitsdefizit-Hyperaktivitätsstörung. (Basierend auf [3])

Diese Ansätze basieren auf einer pädagogischen Grundhaltung, die von einer unbedingten Wertschätzung, Authentizität und Empathie geprägt ist [4]. In Abb. 1 sind die Ziele der SGFK, die jeweils zugeordneten Aufgabenbereiche sowie die Vernetzungsfunktionen der SGFK dargestellt.

Das Ziel „Gesundheitskompetenz erhöhen“ umfasst die Vermittlung von gesundheitsrelevanten Informationen sowie die Förderung der Kompetenzen von Schülerinnen, Schülern und ihren Eltern, gesundheitsrelevante Informationen zu verstehen, zu bewerten und anzuwenden [5]. Das Ziel „Gesundheitsversorgung anbieten“ umfasst pflegerische Tätigkeiten durch SGFK, die darauf abzielen, eine bestehende Erkrankung zu lindern bzw. deren Fortschreiten entgegenzuwirken. Ein wichtiger Bestandteil dieses Ansatzes sind Erste-Hilfe-Maßnahmen, die pflegerische Begleitung von chronisch erkrankten Schülerinnen und Schülern sowie die Betreuung von Kindern mit psychischen Herausforderungen, wie Prüfungsängsten oder Aufmerksamkeitsdefizit-/Hyperaktivitätsstörung(ADHS)-Symptomen [5]. Das dritte Ziel „Gesundheitsförderung umsetzen“ nimmt die salutogenetische Perspektive ein und beschäftigt sich mit gesundheitsfördernden Interventionen. Die Aufgaben umfassen ressourcenstärkende Maßnahmen an Schulen durch Bewegung wie die „Bewegte Pause“ [5], gesunde Ernährung (Schulmensa, Herkunft von Nahrungsmitteln) oder Überprüfung des Raumklimas [5].

Die 3 Ziele sind nicht getrennt voneinander zu sehen. Vielmehr repräsentieren sie unterschiedliche fachliche Perspektiven und Haltungen, die das komplexe und heterogene Tätigkeitsfeld der SGFK verdeutlichen. Von zentraler Bedeutung ist die fachliche Abgrenzung der Tätigkeit der SGFK gegenüber ärztlichen, psychologischen oder sozialpädagogischen Berufsgruppen. SGFK haben die Aufgabe, Schülerinnen und Schüler bei Bedarf an regionale Versorgungs- und Unterstützungsstrukturen weiterzuvermitteln und sie beratend zu begleiten.

Der Artikel gibt einen Überblick über den Stand des Einsatzes von SGFK an deutschen Schulen. Er fasst Rechercheergebnisse zu Modellprojekten zusammen und zeigt, inwieweit diese bereits verstetigt wurden. Zudem werden die Bedeutung von SGFK für die gesundheitliche Versorgung von Kindern und Jugendlichen sowie die internationale Evidenz zur Wirksamkeit ihres Einsatzes dargestellt. Abschließend werden Hindernisse und Möglichkeiten einer flächendeckenden Umsetzung im deutschen Schulsystem diskutiert.

## Einsatz von Schulgesundheitsfachkräften an deutschen Schulen

Neben ihrem Bildungsauftrag stehen Lehrkräfte im Schulalltag vor vielfältigen Herausforderungen, die über die reine Wissensvermittlung hinausgehen [6]. Um diese Herausforderungen zu bewältigen, wurde der Bedarf von interdisziplinären Berufsgruppen längst erkannt [7]. Durch eine Empfehlung im „Strategiepapier zum Nationalen Aktionsplan: Gesundheitskompetenz (NOK)“ 2018 wurde die Bedeutung der SGFK als zusätzliche Berufsgruppe an Schulen unterstrichen [8], indem die SGFK als Ressource benannt wird, die in Bildungsinstitutionen verankert werden solle [8]. Dieses Strategiepapier basiert auf Empfehlungen von Expertinnen und Experten, die zum Ergebnis kommen, dass „die Gesundheitskompetenz so früh wie möglich im Lebenslauf zu [entwickeln sei]“ [8].

Erste Versuche, die SGFK in Deutschland zu etablieren, gab es bereits im Jahr 1908 im Auftrag der Stadt Charlottenburg in Berlin [9]. Die damals sogenannten Schulschwestern (heute Schulgesundheitsfachkräfte) stammten aus einem Kirchenorden und unterlagen den Anordnungen des Schularztes bzw. der Schulärztin [9]. Mit dem Beginn nationalsozialistischer Ideologien kam es zu einer Umstrukturierung des Schul- und Gesundheitssystems, sodass davon auszugehen ist, dass angestellte Schulärzte und Schulärztinnen sowie Schulschwestern Berufsverbote bekamen und durch solche ausgetauscht wurden, die Mitglieder der Nationalsozialistischen Deutschen Arbeiterpartei (NSDAP) oder Meinungsvertreterinnen bzw. Meinungsvertreter waren [9]. Mit dem Ende des 2. Weltkrieges gelang die Reetablierung der Schulschwestern dabei nicht [9]. Erst im Jahr 2015 im Rahmen eines Modellprojektes in Schleswig-Holstein [21] und im Jahr 2016 in Brandenburg und Hessen gelang die Reintegration der Schulschwester bzw. SGFK [11–18].

In allen Bundesländern wurden der Stand von Modellvorhaben sowie Informationen zu ihrer Evaluation und Verstetigung über mehrere Quellenanalysen recherchiert, z. B. schriftliche Anfragen an Kultus- und Gesundheitsministerien, Internet- und Publikationsrecherche sowie persönliche Telefonbefragungen in Gesundheitsämtern. Die Ergebnisse der Recherche sind in Tab. 1 dargestellt. Insgesamt gibt es 8 Bundesländer, die Modellvorhaben bis dato umgesetzt haben. In 7 Bundesländern wurden Modellprojekte wissenschaftlich begleitet, wobei für das Vorhaben in Brandenburg 8 veröffentlichte Studien gefunden wurden [11–18]. Bei einer dieser Untersuchungen handelt es sich um eine Publikation, die einen Peer-Review-Prozess durchlaufen hat [11]; eine weitere, veröffentlicht über einen Peer-Review-Prozess, wird voraussichtlich Ende 2025 publiziert (Abschlussbericht siehe [20]). Die weiteren Ergebnisse der Modellvorhaben liegen als Evaluationsberichte vor, die keinen Peer-Review-Prozess durchlaufen haben.

**Tab. 1:** Übersicht über Modellprojekte zu Schulgesundheitsfachkräften (SGFK) und deren Verstetigung an deutschen Schulen. (Eigene Darstellung basierend auf frei verfügbaren Quellen [10–22])

Bundesland^a^	Modellprojekt						Verstetigung			
	Laufzeit	Förderer	Einsatz der Schulgesundheitsfachkräfte		Evaluation		Förderer/-topf	Einsatz der Schulgesundheitsfachkräfte		Beginn Dauer
			*n*	Schulart/en (*n* Schulen)	Institution	Publikationen (mit/ohne Peer Review)		*n* SGFK	*n* Schulen (GS oder WS) mit SGFK pro BL *N Schulen (*GS oder WS) *pro BL *gesamt (Anteil in %)	
Baden-Württemberg	2021–2024	Projektmittelfond „Zukunft der Jugend“ Eduard-Pfeiffer-Stiftung Unfallkasse Baden-Württemberg Ministerium für Soziales, Gesundheit und Integration	3	GS, WS (5)	Abteilung Gesundheitsförderung und Planung Gesundheitsamt Stuttgart	–/[10]	Kommune Stuttgart^b^	10	13 2523 (0,5 %)^b^	2024 Unbefristet
Berlin	2022–2023	Bezirk Berlin-Lichtenberg	3	GS (6)	–	–/–	Landeshaushalt	9	9 448 (2 %)	2025 k. A.
Brandenburg	2016–2021	Land Brandenburg AOK Nordost Unfallkasse Brandenburg AWO Bezirksverband Potsdam e. V.	18	GS, WS (27)	Universität Lüneburg Charité Berlin Unfallkasse Hessen Unfallkasse Brandenburg Hochschule Mittelhessen	[11]/[12–18]	Einzelne Kommunen/Schulträger (z. B. Cottbus, Brandenburg an der Havel, Neuruppin)	k. A.	k. A.	2021 Unbefristet
Bremen	2018–2020	Senatorin für Gesundheit, Frauen und Verbraucherschutz Gesetzliche Krankenkassen im Land Bremen	7	GS (12)	Privates Institut für angewandte Versorgungsforschung GmbH (inav)	–/[19]	Landeshaushalt	26	26 110 (24 %)	2021 Unbefristet
Hamburg	2020–2025	Verband der Ersatzkassen e. V. Behörde für Schule und Berufsbildung Behörde für Arbeit, Gesundheit, Soziales, Familie und Integration Bezirksamt Hamburg-Nord Unfallkasse Nord	13	GS (18)	–	–/–	Landeshaushalt	11	11 223 (5 %)	2025 Unbefristet
Hessen	2017–2018	Hessische Arbeitsgemeinschaft für Gesundheitsförderung e. V. (HAGE) AOK Sozialministerium	10	GS, WS (10)	Institut für Gesundheits- und Pflegewissenschaft Charité Universitätsmedizin Berlin	[11]/[12, 14]	Landeshaushalt	50	52 7589 (1 %)	2018 Unbefristet
Rheinland-Pfalz	2018–2023	Landeszentrale für Gesundheitsförderung Rheinland-Pfalz Ministerium für Bildung Rheinland-Pfalz	24	GS (24)	Institut für Medizinische Biometrie, Epidemiologie und Informatik, Universität Mainz	–/[20]	Landeshaushalt	26	26 964 (3 %)	2023 Unbefristet
Schleswig-Holstein	2015–2025	Ministerium für Soziales, Gesundheit, Wissenschaft und Gleichstellung Seit 2020 Bildungsministerium	2	GS (2)	Stadt/Gesundheitsdienste Flensburg	[21]/–	k. A.	k. A.	k. A.	k. A.
	Seit ca. 1980	k. A.	k. A.	Kitas, GS, WS der dänischen Minderheit (k. A.)	–	–/[22]	Dänisches Gesundheitsministerium Einzelne Gemeinden in Flensburg	9	46 46 (100 %)	1980 Unbefristet

*SGFK* Schulgesundheitsfachkräfte, *BL* Bundesland, *k.* *A.* keine Angabe, *–* nicht vorhanden, *GS* Grundschulen, *WS* weiterführende Schulen

^a^Die Bezeichnung und Abkürzung der Schulgesundheitsfachkraft unterscheidet sich in den jeweiligen Bundesländern: Baden-Württemberg, Berlin, Brandenburg, Hamburg, Hessen = Schulgesundheitsfachkraft (SGFK); Rheinland-Pfalz, Schleswig-Holstein = Schulgesundheitsfachkraft (SGF); Bremen = Gesundheitsfachkraft Schule (GefaS)

^b^Für das Bundesland Baden-Württemberg waren nur Angaben aus Stuttgart ermittelbar

Nach Beendigung der Modellvorhaben (ausgenommen Schleswig-Holstein) übernahm entweder der Landeshaushalt oder einzelne Kommunen die Finanzierung. In den Bundesländern Baden-Württemberg, Brandenburg, Bremen, Hamburg, Hessen, Rheinland-Pfalz und der dänischen Minderheit in Schleswig-Holstein ist die Verstetigung auf unbegrenzte Zeit finanziert. In den meisten Bundesländern wird eine SGFK pro Schule einkalkuliert. Die Rechercheergebnisse zeigen, dass die Anzahl der finanzierten SGFK sehr gering ist, zum Beispiel mit nur 9 SGFK in Berlin (448 Grundschulen), 26 SGFK in Bremen (110 Grundschulen) und Rheinland-Pfalz (964 Grundschulen), 11 SGFK in Hamburg (223 Grundschulen) und 50 SGFK in Hessen (7589 Grundschulen und weiterführende Schulen). Baden-Württemberg bzw. Stuttgart hat 8 Stellen genehmigt (2523 Grundschulen und weiterführende Schulen im Bundesland). Diese Analyse zeigt somit, dass SGFK an 2 % aller Grundschulen in Berlin, 0,5 % aller Grund- und weiterführenden Schulen in Baden-Württemberg, 24 % aller Grundschulen in Bremen, 5 % aller Grundschulen in Hamburg, 0,7 % aller Grund- und weiterführenden Schulen in Hessen und 3 % aller Grundschulen in Rheinland-Pfalz vertreten sind.

Auf Basis bisheriger Modellprojekte wird in Deutschland ein durchschnittlicher Versorgungsschlüssel von 1:700 Schülerinnen und Schülern empfohlen, welcher in Abhängigkeit von Region, Schultyp und Aufgabenspektrum angepasst werden soll [9]. Internationale Studien zeigen ergänzend, dass die Arbeitszufriedenheit von SGFK eng mit dem Versorgungsschlüssel zusammenhängt [23].

Für die 8 Bundesländer mit Modellprojekten wurden ebenfalls Informationen zu den erforderlichen Qualifikationen der SGFK recherchiert. Voraussetzung für die Qualifikation zur SGFK ist eine abgeschlossene Berufsausbildung im pflegerischen Bereich. Außerdem müssen SGFK in den meisten der 8 Bundesländern eine 3‑jährige Berufserfahrung im Pflegeberuf sowie die Bereitschaft für weiterführende Qualifizierungsmaßnahmen vorweisen (Tab. 2). Der Anspruch, ein standardisiertes Qualifizierungsangebot zu entwickeln, führte zur Erarbeitung eines Curriculums [2], das auf den Ergebnissen einer Machbarkeitsstudie aus Brandenburg und Hessen basiert [18]. Dieses Curriculum orientiert sich an den internationalen Richtlinien der Weltgesundheitsorganisation (WHO) und wurde zu einem modularen Programm weiterentwickelt, das an die Rahmenbedingungen in Deutschland angepasst ist. Es umfasst Online- und Präsenzveranstaltungen und schließt mit einem Zertifikat ab.

**Tab. 2:** Übersicht über die Tätigkeiten der Schulgesundheitsfachkräfte (SGFK) und die geforderten Qualifikationen in den 8 Bundesländern mit Modellprojekten. (Eigene Darstellung basierend auf frei verfügbaren Quellen [2, 5, 10–22])

Bundesland	Tätigkeiten	Geforderte Qualifikation
Baden-Württemberg [10]	Akutversorgung Unterstützung bei chronischen Erkrankungen Förderung der Gesundheitskompetenz Prävention Ansprech- und Vertrauensperson für SuS Vermittlung ins Hilfssystem Initiierung von Aktionen zur Gesundheitserziehung	Abgeschlossene Berufsausbildung als Familien‑, Gesundheits- oder (Kinder‑)KrankenpflegerIn Mindestens 3‑jährige Berufserfahrung Weiterqualifizierung durch Gesundheitsamt und externe Fortbildungen (keine genaueren Angaben verfügbar)
Berlin [47]	Medizinisch-pflegerische Versorgung Akutversorgung Medizinische Pflege Förderung der Gesundheitskompetenz Ansprech- und Vertrauensperson für Schülerinnen und Schüler (SuS) Fokus auf Armutsbekämpfung	Abgeschlossene Berufsausbildung als Pflegefachkraft Berufserfahrung im Bereich Kinderkrankenpflege erwünscht Allgemeine Kenntnisse über Gesundheitsdienstgesetz (GDG), Infektionsschutzgesetz (IfSG), Schulgesetz, Sozialgesetzbuch (SGB) XII
Brandenburg [11–18]	Akutversorgung Gesundheitsförderung und -prävention Früherkennung Netzwerkarbeit Unterstützung bei chronischen Erkrankungen Ansprech- und Vertrauensperson für SuS	Abgeschlossene Ausbildung als Gesundheits- oder (Kinder‑)KrankenpflegerIn 3‑jährige Berufserfahrung Weitere Qualifizierungsmaßnahmen [2]
Bremen [19]	Gesundheitsförderung im Schulalltag (Workshops, Lerneinheiten) Individuelle Beratung und Unterstützung Initiierung und Umsetzung von Gesundheitsprojekten für eine gesunde Schulgemeinschaft Brückenfunktion durch Vernetzung und Zusammenarbeit mit AkteurInnen und gesundheitsfördernden Strukturen im Stadtteil Ansprech- und Vertrauensperson für SuS	Abgeschlossene Ausbildung als Gesundheits- oder (Kinder‑)KrankenpflegerIn Hochschulabschluss im Bereich Public Health Weiterqualifikation und Supervision, Qualifizierungsmodule in den Schulferien [2]
Hamburg [5]	Unterstützung von Familien in schwierigen sozialen Lagen Förderung der Gesundheitskompetenz AnsprechpartnerIn bei SuS mit Adipositas oder psychischen Auffälligkeiten Ansprech- und Vertrauensperson für SuS	Abgeschlossene Ausbildung als Gesundheits- oder (Kinder‑)KrankenpflegerIn Mindestens 3‑jährige Berufserfahrung
Hessen [11, 12, 14]	Akutversorgung Unterstützung bei akuten Beschwerden oder chronischen Erkrankungen Aktive Unterstützung der Maßnahmen des Kinder- und Jugendgesundheitsdienstes (KJGD) Früherkennung Beratung und Unterrichtsbegleitung Eingliederung von SchülerInnen mit Behinderung Kooperation mit anderen Fachkräften Ansprech- und Vertrauensperson für SuS Ansprechperson für den Öffentlichen Gesundheitsdienst (ÖGD)	Abgeschlossene Berufsausbildung als Gesundheits- oder KrankenpflegerIn 3‑jährige Berufserfahrung Bereitschaft für weiterführende Qualifizierungsmaßnahmen [2]
Rheinland-Pfalz [20]	Medizinisch-pflegerische Versorgung Fokus auf der Versorgung, nicht auf Prävention Im Einzelfall auch Maßnahmen wie Hygieneschulungen Ansprech- und Vertrauensperson für SuS	Abgeschlossene Berufsausbildung als KinderkrankenpflegerIn [5] Mindestens 5‑jährige Berufserfahrung [5]
Schleswig-Holstein [21]	Akutmedizinische Versorgung und Beratung Förderung von Gesundheitskompetenz Netzwerkarbeit Koordination Ansprech- und Vertrauensperson für SuS	Abgeschlossene Berufsausbildung als medizinische/r Fachangestellte/r Hochschulstudium Gesundheitsförderung

Aus den bisherigen Ergebnissen der Modellprojekte lassen sich zu verschiedenen Themenbereichen Rückschlüsse auf die Machbarkeit und Wirkung ziehen. So wurde beispielsweise die Wirksamkeit der SGFK auf das Unfallgeschehen an allgemeinbildenden Schulen in Brandenburg und Hessen untersucht [11]. Ergebnisse der Prä-Post-Analyse zeigen, dass an beiden Modellschulstandorten die Zahl der Einsätze von Rettungswagen (RTW) deutlich reduziert werden konnte. In Brandenburg konnte an Grundschulen der Einsatz um 16 % und an Oberschulen um 13 % reduziert werden. An hessischen Gymnasien konnten die Anzahl der RTW-Einsätze um 46 % und an Gesamtschulen um 64 % reduziert werden. Durch den methodischen Vergleich zu Referenzschulen ohne SGFK wurde ermittelt, dass sich die jährliche Einsparung durch eine Reduktion der RTW-Einsätze an Brandenburger Grundschulen auf 120.000 €, an Oberschulen auf 112.000 € belaufen. In Hessen lagen die Einsparungen an Gymnasien bei 189.000 € und an Gesamtschulen bei 515.000 € [11]. Evaluationsergebnisse aus den Modellvorhaben haben vermutlich bereits zur Implementierung von SGFK an deutschen Schulen beigetragen und werden voraussichtlich auch zukünftig deren weitere Verstetigung unterstützen.

Die dargestellte Übersicht zu den Modellprojekten erhebt keinen Anspruch auf Vollständigkeit, da im Rahmen der Recherche möglicherweise nicht alle aktuellen Vorhaben der Bundesländer sowie bereits abgeschlossene oder geplante Verstetigungen erfasst werden konnten. Dies kann unter anderem daran liegen, dass Informationen zu einzelnen Projekten nicht öffentlich zugänglich oder noch nicht dokumentiert sind.

## Gesundheitsbezogene Bedarfe von Kindern und Jugendlichen im Schulsetting

Denken und Lernen sind komplexe Kompetenzen, die nicht nur auf neurobiologische Prozesse im Gehirn zurückzuführen sind, sondern den gesamten Körper einbeziehen und in engem Zusammenhang mit dem sozialen Umfeld stehen [24]. Erfolgreiches Lernen setzt psychisches Wohlbefinden und eine angemessene körperliche Gesundheitsversorgung voraus. Aktuelle Zahlen zur psychischen und körperlichen Gesundheit von Kindern und Jugendlichen verdeutlichen den bestehenden Handlungsbedarf:

### Psychische Auffälligkeiten.

Die Corona-und-Psyche(COPSY)-Studie, die die psychische Gesundheit von Kindern und Jugendlichen von 2017 bis 2024 bewertet hat, zeigt, dass etwa ein Fünftel der Kinder und Jugendlichen aktuell aufgrund verminderter Lebensqualität, psychischer Probleme oder allgemeiner Angstzustände psychisch beeinträchtigt ist [25]. SGFK können eine wichtige Rolle bei der Identifikation und Bewältigung der psychischen Probleme von Kindern und Jugendlichen spielen. Sie tragen zur Früherkennung psychischer Auffälligkeiten bei und können als Bindeglied zwischen Schülerinnen, Schülern, ihren Familien und Fachkräften der psychischen Gesundheitsversorgung fungieren. Dabei unterstützen sie die Weitervermittlung an geeignete Behandlungsangebote und weiterführende Hilfesysteme.

### Chronische Erkrankungen.

Die Zahl an – auch während der Schulzeit – versorgungsbedürftigen Kindern und Jugendlichen steigt in Deutschland. Die Prävalenz von Typ-1-Diabetes im Kindes- und Jugendalter liegt bei etwa 0,3 % [26], Tendenz steigend [27]. Bei kindlichem Asthma liegt die 12-Monats-Prävalenz bei etwa 4,7 % [28]. Unter chronischen Schlafschwierigkeiten leiden laut einer Studie aus dem Jahr 2022 etwa 22 % der 11- bis 17-jährigen Kinder und Jugendlichen [29]. SGFK sind bei der Unterstützung von Kindern mit chronischen Krankheiten durch direkte Pflege und Betreuung von besonderem Wert. Sie koordinieren die Versorgung zwischen Familien und Gesundheitsdienstleistern, verabreichen Medikamente, überwachen den Gesundheitszustand und informieren sowohl Schülerinnen und Schüler als auch das Schulpersonal über spezifische gesundheitsbezogene Bedürfnisse. Dadurch wird sichergestellt, dass Schülerinnen und Schüler mit chronischen Krankheiten sicher am Fachunterricht und außerunterrichtlichen Aktivitäten einer Schule teilnehmen können.

### Gewalterfahrungen.

Laut dem statistischen Bundesamt stieg die Zahl der von häuslicher Gewalt betroffenen Kinder und Jugendlichen 2022 um 4 % im Vergleich zum Vorjahr [30]. Die Zahl der Inobhutnahme von Kindern und Jugendlichen lag 2023 bei 74.600 Kindern – ein neuer Höchststand [30]. Die Gefährdung ging in 73 % der Fälle von einem Elternteil aus und das durchschnittliche Alter des betroffenen Kindes lag bei 8 Jahren. Die Institution Schule könnte „als ein sicherer Ort“ [31] eine zentrale Rolle im Kinderschutz einnehmen [31]. Eine kürzlich veröffentlichte Studie aus Schweden zeigt, dass die SGFK eine zentrale Rolle bei der Identifizierung von Kindeswohlgefährdung spielen kann [32]. Voraussetzungen dafür sind niedrigschwellige Gesprächsmöglichkeiten mit den Schülerinnen und Schülern, ausreichend Zeit zur Vertrauensbildung, die Fähigkeit zur interdisziplinären Zusammenarbeit sowie ein Bewusstsein für mögliche Hemmnisse bei der Offenlegung von Gewalterfahrungen [32]. Eine enge Zusammenarbeit der SGFK mit Schulsozialarbeitenden, die häufig auch als „insoweit erfahrene Kinderschutzfachkräfte“ ausgebildet sind, kann in der Institution Schule zu einer erhöhten Sensibilität und zur Unterstützung der von Gewalt betroffenen Kinder und Jugendlichen führen.

Die genannten gesundheitsbezogenen Bedarfe von Kindern und Jugendlichen im Setting Schule sind exemplarisch zu verstehen und können – wie in Abb. 1 dargestellt – um weitere Krankheits- und Versorgungsbereiche ergänzt werden. Die Relevanz und Notwenigkeit der SGFK an Schulen in Deutschland wird auch in den Evaluationsstudien der Modellvorhaben übereinstimmend bestätigt. Zur evidenzbasierten Bewertung der gesundheitsbezogenen Wirksamkeit können systematische Übersichtsarbeiten und Metaanalysen herangezogen werden, die in peer-reviewten, indexierten Fachzeitschriften veröffentlicht wurden und damit eine Qualitätssicherung und Vergleichbarkeit der Studien gewährleisten.

## Internationale Datenlage zur Wirksamkeit

Die Wirksamkeit von SGFK wird international seit Jahrzehnten bereits an gesundheitsbezogenen Kriterien gemessen. Internationale Studien, überwiegend aus angelsächsischen und skandinavischen Ländern [33], untersuchten die Wirksamkeit von SGFK in Bezug auf die Reduktion krankheitsbedingter Fehltage [34], die Verringerung gesundheitlicher Risikofaktoren wie Adipositas [35] oder Tabakkonsum [36] sowie auf Interventionen zur Behandlung von chronischen Krankheiten wie Asthma oder Diabetes [37]. Eine jüngst veröffentlichte Übersichtsarbeit lieferte eine differenzierte Analyse von 16 systematischen Übersichtsarbeiten (1976 bis 2021 publiziert) bzw. 289 darin enthaltenen Primärstudien (1937 bis 2018 publiziert) zu gesundheitlichen Interventionen durch SGFK [3]. Die Wirksamkeit ihrer Interventionen wurde hinsichtlich ihres Einflusses auf Erkrankungen, wie Asthma, Diabetes, Risikoverhalten oder Verhaltensauffälligkeiten bewertet, indem Ergebnisse der Primärstudien zusammengefasst und anhand ihrer Studiendesigns und methodischen Qualität evaluiert wurden [3]. Die Ergebnisse zeigen, dass SGFK die Gesundheit von Kindern mit Asthma [38] und Diabetes [39] signifikant verbessern können. Als weitere relevante Wirkungsfelder der SGFK ergab das Literaturreview die Reduktion von Risikoverhaltensweisen, Verbesserung von Depressions- und Angstsymptomen sowie die Verbesserung von Hygieneverhalten bei Schülerinnen und Schülern [3]. Von den 289 Primärstudien waren ca. 5 % (16 Studien) von ausreichender methodischer Qualität, d. h., es handelte sich um randomisierte kontrollierte Studien oder Beobachtungsstudien mit geringem Risiko systematischer Verzerrungen [3].

Eine zweite Übersichtsarbeit beschäftigte sich mit der Wirksamkeit von SGFK bei sozial benachteiligten Schülerinnen und Schülern [40]. Der Aspekt der sozialen Benachteiligung spielt eine zentrale Rolle in der Arbeit der SGFK, da benachteiligte Kinder und Jugendliche nachweislich höheren gesundheitlichen Belastungen ausgesetzt sind [41] als Gleichaltrige, die sozial besser gestellt sind. Die Ergebnisse der Übersichtsarbeit [40] deuten darauf hin, dass SGFK-basierte Interventionen positive Effekte in mehreren Bereichen zeigen – darunter Verhaltensauffälligkeiten (z. B. Hyperaktivität, interaktionelle Probleme [42]), psychische Symptome (z. B. Depression [43]) sowie die Asthma-Symptomatik und Inhalationstechnik [44]. Einer bereits 1980 publizierten Studie zufolge neigten Kinder mit getrennt lebenden Eltern eher dazu, die SGFK aufzusuchen, als Schülerinnen und Schüler aus intakten Familien, wobei Mädchen häufiger als Jungen die SGFK besuchten [45]. Alle Studien liefern Hinweise auf die Wirksamkeit von SGFK für sozial benachteiligte Schülerinnen und Schüler.

## Diskussion

Vor dem Hintergrund der dargestellten empirischen Datenlage ist davon auszugehen, dass SGFK eine wichtige Ressource an deutschen Schulen darstellen. Durch die Etablierung der SGFK bekommt Gesundheit im Kontext Schule einen neuen Fokus. Die Bedeutung von Gesundheit für erfolgreiches Lernen wird zunehmend diskutiert und die Frage der Umsetzung von „gesund zu lernen“ rückt auch im politischen Diskurs immer weiter in den Mittelpunkt, insbesondere in Zusammenhang mit Bildungschancen und Bildungsgerechtigkeit.

Modellprojekte in 8 Bundesländern wurden bereits verstetigt. Weitere Implementations- und Evaluationsforschung ist bei diesen Bemühungen jedoch noch erforderlich, insbesondere aber eine Verbesserung der Sichtbarkeit der deutschen Evaluationsergebnisse in Fachpublikationen mit Peer Review. Nur so kann eine Evidenzbasierung des Einsatzes von SGFK an deutschen Schulen gelingen und politische Entscheidungsträger motiviert werden das Angebot auch in Deutschland auszuweiten. Die Verstetigung von SGFK in den Bundesländern Baden-Württemberg, Bremen, Hamburg, Hessen und Rheinland-Pfalz zeigt erste Fortschritte und hat positive Resonanz erfahren. Dennoch zeigen Analysen, dass Modellprojekte, wie in Brandenburg, Hamburg und Rheinland-Pfalz, den Stellenumfang weitgehend beibehalten und die Finanzierung auf vergleichbarem Niveau fortsetzen, nicht aber skalieren. Ermutigende Einzelbeispiele gibt es dennoch: In Hessen gelang es den Stellenumfang von 10 SGFK im Modellprojekt auf 50 SGFK nach Abschluss des Modellprojektes zu skalieren. In Baden-Württemberg finanzierte die Kommune Stuttgart 8 SGFK-Stellen nach Abschluss des Modellprojektes mit 3 SGFK. Insgesamt zeigen die Ergebnisse aber, dass eine bedarfsgerechte Ausweitung der SGFK an Grund- und weiterführenden Schulen bisher schleppend voranschreitet. Ein möglicher Grund dafür könnten die hohen finanziellen Kosten bei Etablierung der SGFK für die Schulen selbst sein, die sich finanziell beteiligen und die räumlichen Voraussetzungen schaffen müssen.

Insgesamt ist die Etablierung von SGFK an deutschen Schulen trotz positiver nationaler Evaluationsergebnisse und internationaler Wirksamkeitsnachweise noch gering: In Bremen haben 75 % aller Grundschulen keine SGFK, in Baden-Württemberg 90 %, in Rheinland-Pfalz 97 %, in Berlin 98 % und in Hessen 99 %. Eine Hürde, die die umfassendere Verstetigung des SGFK-Konzeptes erschweren könnte, sind Befürchtungen, die Etablierung der SGFK könne zu einer Zunahme des Fachkräftemangels in anderen Pflegeberufen führen. Dem ist die Steigerung der Attraktivität des Pflegeberufs zu entgegnen [46]. Die SGFK kann Schülerinnen und Schülern einen neuen Zugang zum Pflegeberuf eröffnen und das oft negative Berufsbild positiv verändern. Darüber hinaus zeigte das Modellprojekt in Bremen, dass Pflegekräfte, die nicht berufstätig waren, beispielsweise aufgrund der Unvereinbarkeit von Schichtarbeit mit familiären Verpflichtungen, durch die Tätigkeit als SGFK wieder in den Arbeitsmarkt integriert werden konnten.

Um die Umsetzung von SGFK an deutschen Schulen weiter zu fördern, sind eine verlässliche finanzielle Regelung und auf politischer Ebene eine neue Schwerpunktsetzung auf „Bildung und Gesundheit“ erforderlich. Das Motto „gesund lernen“ gelangte durch eine Vielzahl erfolgreicher Modellprojekte, aber auch internationale Forschungsarbeiten [3] insbesondere in den letzten Jahren verstärkt in den Fokus politischer Akteurinnen und Akteure. Viele Schritte hinsichtlich einer Standardisierung des Berufs wurden bereits gemacht, beispielsweise durch die Etablierung eines Curriculums [2], gleichzeitig fehlt es an einer landesgesetzlichen Grundlage, die je nach organisatorischer Anbindung im Recht des Öffentlichen Gesundheitsdienstes (ÖGD) oder im Schulrecht anzusiedeln ist [2]. Mit Blick auf die Anzahl beschäftigter SGFK wird es notwendig sein, entsprechende finanzielle Mittel der jeweiligen Landeshaushalte bereitzustellen, um SGFK flächendeckender einzusetzen. Dabei sind gesundheitsökonomische Evaluationen zum Mehrwert des Einsatzes von SGFK im Sinne langfristiger Einsparungen von Gesundheitsfolgekosten für eine begründete Entscheidung zur Ausweitung des Konzepts an Schulen notwendig.

## Ausblick

Die gesundheitsfördernde und pflegerische Versorgung durch SGFK wird an deutschen Schulen noch nicht ausreichend gewährleistet. Aktuell werden Schülerinnen und Schüler mit Asthma oder Diabetes, aber auch Kinder mit Kopf- und Bauchschmerzen oder Allergien von Lehrkräften nach Hause geschickt bzw. die Sorgeberechtigten um deren Abholung gebeten. Chronische Erkrankungen erfordern kontinuierliche Betreuung sowie fundiertes Wissen im Umgang mit der eigenen Gesundheit. SGFK setzen genau hier an: Sie zeigen Kindern und Jugendlichen, wie sie ihren Blutzucker richtig messen oder ihre Inhalationstechnik verbessern können. Darüber hinaus gibt es Schülerinnen und Schüler, die zusätzlichen Unterstützungsbedarf haben – Unterstützung, die Lehrkräfte weder leisten dürfen noch können. Zwar wurden in einigen Bundesländern bereits SGFK, die in Modellvorhaben an Schulen tätig waren, unbefristet weiterbeschäftigt, dies macht jedoch nur einen Bruchteil der Schulen in Deutschland aus. Von einer „Regelversorgung“ sind wir noch entfernt, da Zuständigkeiten, Stellenschlüssel, Ausbildungsinhalte und -voraussetzungen, Anbindungen und gesetzliche Grundlagen noch verbindlich vereinbart werden müssen und letztlich auch die Finanzierung langfristig geregelt werden muss. Gleichzeitig wird die SGFK zunehmend als wichtige Ressource zur Betreuung chronisch erkrankter Schülerinnen und Schüler, zur besseren Bildungsgerechtigkeit auch für körperlich und/oder psychisch belastete Kinder und als wichtige Ressource zur Verbesserung der Gesundheitskompetenz gesehen.

Die regelhafte Einführung der SGFK an allen Schulen in Deutschland erfordert eine sorgfältige Planung innerhalb der Schulen und natürlich den politischen Willen. Schul- und gesundheitspolitische Entscheidungsträger können bei ihrer Entscheidungsfindung durch methodisch hochwertige Wirksamkeitsevaluationen, insbesondere im Rahmen von randomisiert kontrollierten Studien (RCT), unterstützt werden. Ergänzend liefern Metaanalysen internationaler Wirksamkeitsstudien sowie gesundheitsökonomische Analysen zum Return on Investment wichtige Evidenzgrundlagen für fundierte politische Entscheidungen.

## Data Availability

Alle Informationen sind im Manuskript enthalten.
