# Erratum zu: Schulgesundheitsfachkräfte in Deutschland – Modellprojekte und Verstetigungen

**DOI:** 10.1007/s00103-026-04221-x

**Published:** 2026-03-16

**Authors:** Franka Metzner-Guczka, Susanne Heumann-Schoop, Daniel Mays, Silke Pawils

**Affiliations:** 1 Institut und Poliklinik für Medizinische Psychologie, Universitätsklinikum Hamburg-Eppendorf, Martinistr. 52, 20246 Hamburg, Deutschland; 2 Fachbereich Erziehungswissenschaft, Universität Bielefeld, Bielefeld, Deutschland; 3 Professur für Pädagogik im Förderschwerpunkt Emotionale und soziale Entwicklung, Institut für Sonderpädagogik, Fakultät I, Pädagogische Hochschule Freiburg, Freiburg im Breisgau, Deutschland

**Erratum zu:**

**Bundesgesundheitsbl 2025**

10.1007/s00103-025-04104-7

In diesem Artikel hätte in dem Satz beginnend mit ‚Baden-Württemberg bzw. Stuttgart hat …‘ der Wert ‚258 Stellen‘ richtigerweise ‚8 Stellen‘ sowie in dem Satz beginnend mit ‚In Baden-Württemberg finanzierte die Kommune Stuttgart …‘ der Wert ‚258 SGFK-Stellen‘ richtigerweise ‚8 SGFK-Stellen‘ lauten sollen.

Der Originalbeitrag wurde korrigiert.

